# Supplementary material for: Splice-Junction-Based Mapping of Alternative Isoforms in the Human Proteome
Source: Cell Rep. Author manuscript; Available in PMC 2020 Jan 15. (PMC6961840; doi:10.1016/j.celrep.2019.11.026)
Supplement: 2 [file NIHMS1546469-supplement-2.zip › DF1/PXD010154/Heart/Heart_5_TTN_TPVQEEVIEVKVPEVPK.pdf]

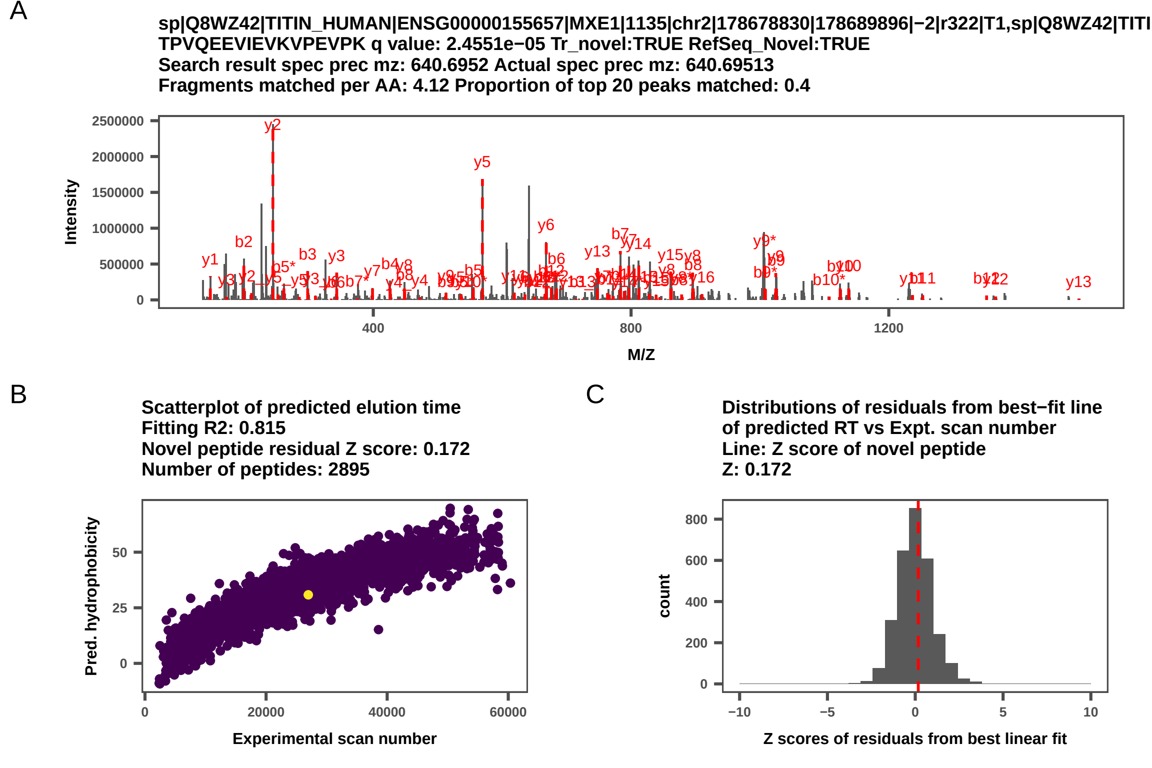
A
sp|Q8WZ42|TITIN_HUMAN|ENSG00000155657|MXE1|1135|chr2|178678830|178689896|−2|r322|T1,sp|Q8WZ42|TITI
TPVQEEVIEVKVPEVPK q value: 2.4551e−05 Tr_novel:TRUE RefSeq_Novel:TRUE
Search result spec prec mz: 640.6952 Actual spec prec mz: 640.69513
Fragments matched per AA: 4.12 Proportion of top 20 peaks matched: 0.4
Intensity
2500000
2000000
1500000
1000000
500000
0
M/Z
400
800
1200
y2
y5
y6
y1
b2
b3
y3
b7
y14
y13
y15
y9*
b10*
y13
B
Scatterplot of predicted elution time
Fitting R2: 0.815
Novel peptide residual Z score: 0.172
Number of peptides: 2895
Pred. hydrophobicity
50
25
0
Experimental scan number
0
20000
40000
60000
C
Distributions of residuals from best−fit line
of predicted RT vs Expt. scan number
Line: Z score of novel peptide
Z: 0.172
count
800
600
400
200
0
Z scores of residuals from best linear fit
−10
−5
0
5
10
